# A Novel Physiological Glycosaminoglycan-Deficient Splice Variant of Neuropilin-1 Is Anti-Tumorigenic *In Vitro* and *In Vivo*

**DOI:** 10.1371/journal.pone.0165153

**Published:** 2016-10-31

**Authors:** Céline Hendricks, Johanne Dubail, Laura Brohée, Yves Delforge, Alain Colige, Christophe Deroanne

**Affiliations:** Laboratory of Connective Tissues Biology, Tour de Pathologie, GIGA-Cancer, University of Liège, Sart-Tilman, Belgium; University of South Alabama Mitchell Cancer Institute, UNITED STATES

## Abstract

Neuropilin-1 (NRP1) is a transmembrane protein acting as a co-receptor for several growth factors and interacting with other proteins such as integrins and plexins/semaphorins. It is involved in axonal development, angiogenesis and cancer progression. Its primary mRNA is subjected to alternative splicing mechanisms generating different isoforms, some of which lack the transmembrane domain and display antagonist properties to NRP1 full size (FS). NRP1 is further post-translationally modified by the addition of glycosaminoglycans (GAG) side chains through an O-glycosylation site at serine_612_. Here, we characterized a novel splice variant which has never been investigated, NRP1-Δ7, differing from the NRP1-FS by a deletion of 7 amino acids occurring two residues downstream of the O-glycosylation site. This short sequence contains two aspartic residues critical for efficient glycosylation. As expected, the high molecular weight products appearing as a smear in SDS-PAGE and reflecting the presence of GAG in NRP1-FS were undetectable in the NRP1-Δ7 protein. NRP1-Δ7 mRNA was found expressed at an appreciable level, between 10 and 30% of the total NRP1, by various cells lines and tissues from human and murine origin. To investigate the biological properties of this isoform, we generated prostatic (PC3) and breast (MDA-MB-231) cancer cells able to express recombinant NRP1-FS or NRP1-Δ7 in a doxycycline-inducible manner. Cells with increased expression of NRP1-Δ7 were characterized *in vitro* by a significant reduction of proliferation, migration and anchorage-independent growth, while NRP1-FS had the expected opposite “pro-tumoral” effects. Upon VEGF-A_165_ treatment, a lower internalization rate was observed for NRP1-Δ7 than for NRP1-FS. Finally, we showed that NRP1-Δ7 inhibited growth of prostatic tumors and their vascularization *in vivo*. This report identifies NRP1-Δ7 as a splice variant displaying anti-tumorigenic properties *in vitro* and *in vivo*, emphasizing the need to consider this isoform in future studies.

## Introduction

The neuropilin-1 (NRP1) gene is located on chromosome 10p12 and composed of 17 exons [1]. It encodes a transmembrane protein involved in axonal development, angiogenesis and cancer progression [2]. It acts as a co-receptor for several growth factors including some variants of VEGF, EGF, HGF, FGF, TGF-β and PDGF [3–7], and it also interacts with other transmembrane proteins such as integrins and plexins/semaphorins [8, 9].

NRP1 can be considered as a proteoglycan since it can be modified by the addition of glycosaminoglycan side chains [10–12]. Serine_612_ has been identified as the site of O-glycosylation where long chains of oligosaccharides assemble on the protein. The nature of the chains attached on NRP1 depends on the cell type. In smooth muscle cells, NRP1 is predominantly glycosylated with chondroitin sulfate while it is derivatized with heparan sulfate in endothelial cells [12]. The number and the nature of the glycosaminoglycans linked to a protein strongly modulate its function. This has also been observed for NRP1. Mutation of Ser_612_ into Ala prevents O-linked glycosylation and markedly affects the biological functions of NRP1 [10, 13]. The suppression of the glycosaminoglycan side chains by enzymatic digestion similarly results in altered NRP1-driven cell invasion [10].

**Fig 1:**
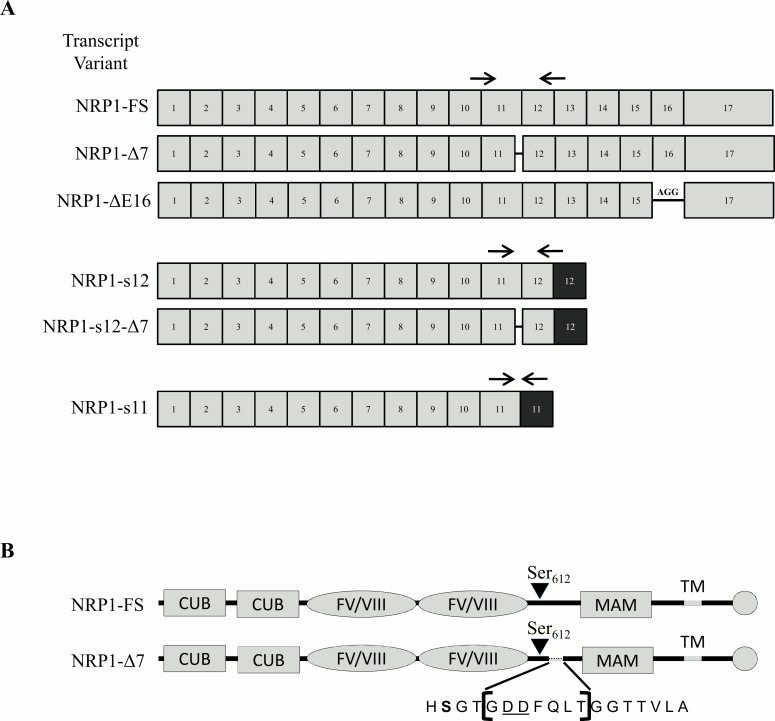
**(A) Schematic representation of the main known and putative neuropilin-1 splice variants**. The exons of the neuropilin-1 isoforms are depicted by grey boxes while the black boxes correspond to intronic sequences retained into some splice variants. Deletions are represented by a black line. The boxes are not scaled to the size of the exon. The position of the primers used to co-amplify the NRP1 variants are indicated with black arrows. **(B) Schematic representation of the protein domains of NRP1-FS and NRP1-Δ7.** The deleted 7 amino acids sequence in NRP1-Δ7 in the vicinity of Ser_612_ is shown between brackets.

The full size NRP1 (NRP1-FS, 923 aa) is known as a pro-angiogenic and pro-tumorigenic factor [13, 14]. Alternative splicing mechanisms occurring on its primary transcript generate different protein isoforms with sizes varying from 551 to 907 amino acids [1] (Fig 1). Four variants (NRP1-s11, s12, sIII and sIV) lacking the transmembrane domain are soluble and able to bind VEGF-A_165_ but exert anti-angiogenic and anti-tumorigenic effects by sequestration of NRP1 binding growth factors and competition with their signaling pathways [15, 16]. NRP1-ΔE16, resulting from the skipping of exon 16 and its replacement by the “AAG” Arg triplet, does not display any functional difference as compared to the NRP1-FS [17].

In the present work, we have identified and studied a so far uncharacterized splice variant of NRP1. Although this variant is quite abundant (10 to 30% of the total amount of NRP1), it was never specifically reported before, most probably because it differs from the NRP1-FS variant by the absence of only 7 amino acids, which prevents its identification on SDS-PAGE. This deletion is located two residues downstream of the O-glycosylation site on serine_612_ and contains two aspartic residues that are critical for efficient glycosylation [18]. We have therefore determined if the modification of the consensus sequence around Ser_612_ affected the glycosylation status and the functions of this new NRP1 variant.

## Materials and Methods

### Reagents and Cells

Doxycyline hyclate (D9891) was from Sigma-Aldrich (USA). Rabbit monoclonal anti-neuropilin-1 (ab81321) was from Abcam (UK). Rabbit polyclonal anti–extracellular signal-regulated kinase (ERK1/2) (M-5670) and mouse monoclonal anti-FLAG (F1804) were from Sigma-Aldrich. Rabbit polyclonal anti-Tet-repressor antibody (TET01) was from MoBiTec (Germany). The secondary horseradish peroxidase–conjugated rabbit anti-mouse IgG (P0260) and swine anti–rabbit IgG (P0217) were from DAKO (Belgium). PC3 human prostate adenocarcinoma cells were cultured in F-12 Kaighn's medium (Invitrogen, Belgium) supplemented with 7% fetal bovine serum (FBS) (Lonza, Belgium). MDA-MB-231 human breast adenocarcinoma cells were cultured in DMEM (Lonza) supplemented with 10% fetal bovine serum. Human skin fibroblasts, HSMC (human smooth muscle cells), HUVEC (human umbilical vein endothelial cells), HT1080 (human fibrosarcoma cells) and MCF7 (human breast adenocarcinoma cells) were cultured in DMEM supplemented with 10% FBS, MO59J (human malignant glioblastoma cells) were cultured in DMEM/F12 (Lonza) supplemented with 10% FBS and OCI-LY-19 (human lymphoma cells) were cultured in RPMI 1640 (Lonza) supplemented with 10% FBS. Murine 4T1, LLC, NIH3T3 and B16F10 were cultured in DMEM supplemented with 10% FBS. RNA from human normal breast, placenta and skin were obtained from the Surgery, Obstetrics and Gynecology Department and from the Dermatology Department. Mouse RNA was purified from 8-weeks old C57Bl6 mice.

### Western Blotting

Cells were lysed in Laemmli buffer and proteins were separated by polyacrylamide gel electrophoresis. Proteins were transferred to a PVDF Transfer Membrane (NEN Life Science Products, USA) which was then blocked for 1 hour with 3% dry milk in PBS-0.05% Tween 20 and incubated for 4h with the primary antibody. After 3 washings, membranes were incubated with the secondary horseradish peroxydase-conjugated antibody for 1h, and revealed by chemoluminescence using home-made ECL acquired with the ImageQuant LAS4000 device (GE Healthcare, USA). The membranes were re-probed with anti-ERK1/2 antibodies used as a control for protein loading.

### RT-PCR

Total RNA was purified from cells with the High Pure RNA Isolation Kit (Roche, Switzerland) and from tissues with Ambion kit using TRIzol reagent (ThermoFisher, USA), following the instructions of the manufacturer. Reverse Transcription-Polymerase Chain Reaction was performed using Tth DNA Polymerase kit (Roche). The primers 5’-GCTGTGAAGTGGAAGCCCCTACA-3’ (forward) and 5’-GGTCTTGTGAGAGCCCCAGCCA-3’ (reverse) were used to amplify NRP1-FS and NRP1-Δ7 in human samples. The primers 5’-GTGGATGAGTGTGACGACGACCA-3’ (forward) and 5’-GAGAGCCCCAGCCAAACTCGCA-3’ (reverse) were used to amplify NRP1-FS and NRP1-Δ7 in murine samples. The amplification products of the two isoforms were separated on a polyacrylamide gel and quantified. Primers 5’-CTTGGTGGGATTGCTGTGGATGAC-3’ (forward) and 5’-GGGCACTCATGGCTATGATGGTGA-3’ (reverse) were used to amplify NRP1-ΔE16. Primers 5’-GGTGGATGAATGTGATGACGACCA-3’ (forward) and 5’-GTCACATTTCGTATTTTATTTGATACCTGA-3’ (reverse) were used to amplify NRP1-s12. Primers 5′-GTTCACCCACTAATAGGGAACGTGA-3′ (forward) and 5′-GATTCTGACTTAGAGGCGTTCAGT-3′ (reverse) were used to amplify human and mouse 28S rRNA. Primers 5’-TCCAGCTCTCTCCACGCGATTCA-3’ (forward) and 5’-ATGACCGTGGGCTTTTCTGTGGC-3’ (reverse), specific of human NRP1, were used in the *in vivo* experiments.

### RT-qPCR

RT-qPCR was performed as previously described [19]. One μg of total RNA was reversed transcribed using SuperScript III Reverse Transcriptase (Invitrogen). Primers 5’-GCTGTGAAGTGGAAGCCCCTACA-3’ (forward) and 5’-TGCCACCTGTGAGCTGGAAGTCA-3’ (reverse) were used to specifically amplify NRP1-FS in human cells and tissues. Primers 5’-GCTGTGAAGTGGAAGCCCCTACA-3’ (forward) and 5’-TGGTGCCACCTGTTCCACTGTG-3’ (reverse) are specific for human NRP1-Δ7. In mice, 5’-GGCTGTGAAGTGGAAGCACCTAC-3’ (forward) and 5’-TGCCTCCTGTGAGCTGGAAGTCA-3’ (reverse) amplified NRP1-FS whereas 5’-GGCTGTGAAGTGGAAGCACCTAC-3’ (forward) and 5’-TGGTGCCTCCTGTGCCACTGTG-3’ (reverse) amplified NRP1-Δ7. Primers specific for human and mouse GAPDH were described previously [20]. Real-time qPCR was performed in a final volume of 20 μl containing 2 μl cDNA (corresponding to 10 ng of total RNA), 300 nM of each primer and 10 μl qPCR MasterMix Plus for SYBR green assays (Eurogentec, Belgium) in the StepOne Real-Time PCR system (Applied Biosystems, Belgium). The polymerase was activated at 95°C for 3 min and the PCR conditions for amplification were 15 sec at 95°C, 20 sec at 66°C and 20 sec at 72°C. The results were analyzed with the StepOne Software. qPCR amplifications were performed in triplicate. The amplification efficiencies of both variants were close to 100% and not significantly different. The relative expression of the two variants was calculated using the formula 2^(-ΔΔCt)^. The specificity of the amplification products was assessed by gel electrophoresis.

### Deep Sequencing

MCF7 were isolated and prepared for next-generation sequencing analysis, as described in a previous publication [20]. The average number of reads approached or exceeded 20 millions. Alignment of transcripts to the genome indicated that 16733 genes were expressed in the sample. Detailed protocol and sequencing data are available in the ArrayExpress database (www.ebi.ac.uk/arrayexpress) under accession number E-MTAB-5104.

### Generation of PC3 and MDA-MB-231 Cells Expressing NRP1-FS, NRP1-Δ7, FLAG-NRP1-FS and FLAG-NRP1-Δ7 in a Doxycycline-Dependent Way

MDA-MB-231 cells were transfected with pcDNA6/TR (Invitrogen) and selected in medium supplemented with blasticidin (4 μg/ml). One clone expressing a high level of tetracycline repressor (MDA-MB-231/TR) was isolated. PC3 cells expressing a high level of tetracycline repressor (PC3/TR) were described previously [19]. The entire sequences of NRP1-FS and NRP1-Δ7 were amplified by RT-PCR, cloned into pcDNA4/TO (Invitrogen), and sequenced. FLAG-tagged NRP1-FS and NRP1-Δ7 were generated by PCR-based approach by introducing (between the codon encoding Lys26 and the codon encoding Cys27) the FLAG sequence (AspTyrLysAspAspAspAspLys) by using the primers 5’-GACTACAAGGATGACGATGACAAGTGTGGCGATACTATAAAAATTGAAAGC-3’ and 5’-CTTGTCATCGTCATCCTTGTAGTCTTTATCGTTGCGAAAAGCGCCGGC-3’. PC3/TR and MDA-MB-231/TR were transfected with pcDNA4/TO/NRP1-FS, pcDNA4/TO/NRP1-Δ7 or pcDNA4/TO/FLAG-NRP1-FS, pcDNA4/TO/FLAG-NRP1-Δ7 and selected in medium supplemented with blasticidin (1 μg/ml) + Zeocin (200 μg/ml) for PC3 cells and blasticidin (2 μg/ml) + Zeocin (400 μg/ml) for MDA-MB-231 cells. Several clones expressing in a doxycycline-dependent way NRP1-FS, NRP1-Δ7, FLAG-NRP1-FS or FLAG-NRP1-Δ7 were isolated and used in this study.

### Immunoprecipitation

Cells were chilled on ice and lysed in buffer containing 1% Igepal, 150 mM NaCl, 20 mM Tris/HCl pH 7.4, 0.1mM AEBSF and 4 μg/ml aprotinin. Lysates were centrifuged for 10 min at 16000g at 4°C. Supernatants were incubated with 1 μg/ml rabbit anti-NRP1 or anti-FLAG antibodies. After 2h of incubation at 4°C, 30 μl of protein A–Sepharose beads (GE Healthcare, UK) were added for 1h to capture immune complexes. Beads were washed four times in ice-cold lysis buffer and boiled in 60 μl of SDS–PAGE lysis buffer. The samples were subjected to western blotting as described above.

### Scratch Assay

A suspension of 50 000 PC3 or 35 000 MDA-MB-231, each inducible for NRP1-FS or -Δ7, was seeded into culture-inserts (#80209, Ibidi, Germany) with or without 200 ng/ml doxycycline in the medium. This type of insert allows the creation of a homogeneous 500 μm width cell-free space in the middle of a confluent cell monolayer. Twenty-four hours after seeding, the inserts were removed and pictures by phase-contrast microscopy of the resulting scratches were taken at T0 (2 pictures/insert, 6 inserts/condition and experiment for each tested clone was performed at least twice). PC3 and MDA-MB-231 were cultured for a further 24h and 8h, respectively. Cell migration was evaluated by measuring the cell-free surface at the beginning of the experiment (T0) and the surface covered by the cells at the end of the experiment by using ImageJ Software (National Institute of Health, USA). Results are expressed as the percentage of wound closure in each condition.

### Proliferation Assay

Cells were seeded in 24 wells costar plates (7500 cells/well) and allowed to attach overnight (PC3) or for 2h (MDA-MB-231). Half of the cultures were supplemented with doxycycline (day 0) and cells were collected after 1, 3 and 5 days (PC3) or after 1, 2 and 3 days (MDA-MB-231). DNA content was assayed by a fluorimetric technique using bisbenzimide and a microplate Gemini EM spectrofluorimeter (Molecular Devices, USA).

### Cell Apoptosis

PC3/NRP1-FS and PC3/NRP1-Δ7 cells were cultured in absence and presence of doxycycline and apoptosis was evaluated by FACS after annexin V–FITC and propidium iodide staining, as previously described [19] using a FACSCanto II double LASER flow cytometer (UV, 488 nm, 633 nm) (BD Biosciences, Belgium). Data were analyzed using FACSDiva Software (BD Biosciences).

### Soft Agar Assay

Anchorage-independent growth was determined by soft agar assay [21]. A total of 2 × 10^3^ PC3 cells or of 5 × 10^3^ MDA-MB-231 cells overexpressing NRP1-FS or NRP1-Δ7 in a doxycycline-dependent way were plated in 60-mm dishes in growth medium containing 0.3% agar, on top of a 0.6% agar gel. The overexpression of the transgene was induced by addition of 1 μg/ml doxycycline. After 21 days, colonies were counted in the whole dishes using an inverted phase-contrast microscope. Results are expressed as the mean number ± SD of two independent experiments performed in quadruplicate.

### VEGF Treatment of PC3/FLAG-NRP1-FS and PC3/FLAG-NRP1-Δ7

Cells were serum starved and treated with doxycycline during 24h. Recombinant VEGF-A (isoform 165 at 37 ng/ml) [4] was added in some cultures for 10 minutes. After fixation with 4% paraformaldehyde in PBS for 15 min and permeabilization with 0.25% Triton X-100 in PBS for 10 min, the samples were blocked with 10% bovine serum albumin in PBS for 30 min before incubating for 2h with a monoclonal mouse anti-FLAG (1:500 in PBS). After three washings, the samples were incubated for 1h with a goat anti-mouse Alexa Fluor 555-conjugated antibody (1:500 in PBS) and counterstained with DAPI (2.5 μg/ml). Fluorescent labeling was assessed by using an epifluorescent Nikon TiS microscope. For each cell, a Z stack was performed by using NIS Elements software. By using ImageJ software, the fluorescence was measured in the whole cell (membrane-associated and intracellular vesicles) and in a zone free of vesicles. The maximum pixels value in this vesicle-free zone was taken as the threshold beyond which fluorescence was considered to be associated with intracellular vesicles. The pixels values above the threshold measured in the whole cell were summed and expressed in % of the total fluorescence.

### Tumor Xenograft

Experimental protocol was approved by the local ethical committee (University of Liège, approval document n°1293). Nude mice (6 week-old, Charles River Laboratories, USA) received an intraperitoneal injection of 60 mg/kg Nembutal before to be subcutaneously injected in each flank with 2 x 10^6^ PC3 conditionally expressing NRP1-Δ7. Control mice had access to regular water while doxycycline (2 mg/ml) was added in drinking water of induced mice. The general condition of mice was monitored daily by visual examination during the entire experiment and tumor volume was evaluated ((length x width x thickness) x π/6, as determined using a caliper). Signs of pain were never observed and the calculated tumor volumes remained always largely below 1 cm^3^ which is considered as the upper acceptable limit for subcutaneous tumors. After 7 weeks, mice were sacrificed by cervical dislocation followed by decapitation and the collected tumors were photographed, dissected, measured and weighed. Fragments of tumors were then used for immunohistological analyses and RNA extraction.

### Immunohistological Analysis of Tumors

Antigen retrieval was performed on 5 μm sections of PC3 tumors by heating at 126°C for 11 minutes in Target Retrieval Citrate buffer (pH 6, Dako, Denmark). After blocking endogenous peroxydases and non-specific antigenic sites (Universal Blocking Reagent, Biogenex, USA), sections were incubated with primary anti-CD31 antibodies (diluted 1:150 in BSA/PBS 1%) for 1h, followed by biotinylated secondary antibodies (diluted 1:300) for 30 minutes and with HRP-conjugated streptavidin (diluted 1:500) for 30 minutes. Signal was amplified by an incubation in Biotinylated Tyramide (diluted 1:100, Perkin Elmer, USA) for 10 minutes, followed by additional 30 minutes in HRP-conjugated streptavidin. Staining was performed with AEC+ High Sensitivity Chromogen (Dako, Denmark) followed by a counterstaining with hematoxylin. Stained sections were scanned with a NanoZoomer slide scanner (Hamamatsu Photonics, Japan) and quantification was performed using ImageJ software (National Institute of Health, USA). Tumor vascularization was quantified both as the number of blood vessels per unit surface and as the % of surface covered by blood vessels.

### Statistical Analyses

Statistical analyses were performed using U Mann-Whiney test or by an Anova test (GraphPad Prism software, GraphPad Sofware, La Jolla, CA, USA) with p ≤ 0.05 considered as significant.

## Results

### Characterization of a Novel Variant of NRP1: NRP1-Δ7

**Fig 2:**
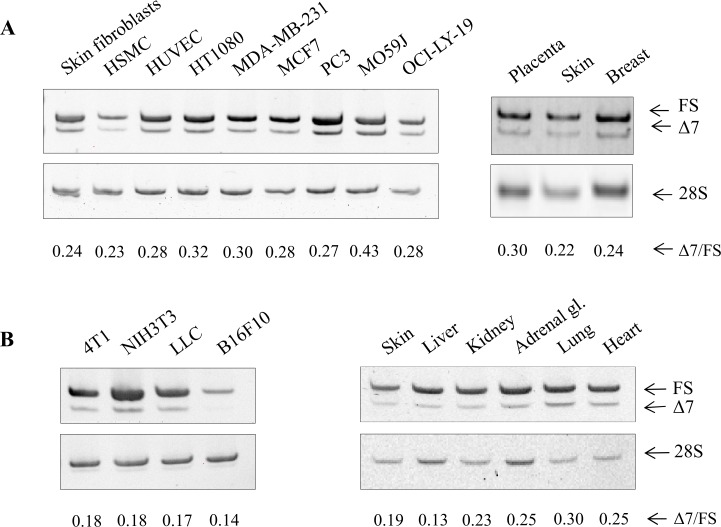
NRP1-Δ7 is expressed in various cell types and tissues. RT-PCR analysis was performed on total RNA extracted from cells and tissues from human (A) and mouse (B) origin as detailed in Materials and Methods. The higher molecular weight product of 233bp is amplified from NRP1-FS (FS) while the lower molecular weight product of 212bp is amplified from NRP1-Δ7 (Δ7). 28S rRNA amplification was used as control for RT-PCR. Quantifications of NRP1-Δ7 and NRP1-FS were also performed by RT-qPCR on the same RNA. The ΔCt for each isoform and their relative abundance are shown in S2 Fig. Only the calculated ratios (Δ7/FS) are provided here.

During an exploratory experiment aiming at determining the expression of the different splice variants of NRP1, several primer pairs were designed and used, including primers at the exon10/exon11 (forward) and exon12/exon13 (reverse) junctions (Fig 1). In addition of the expected 233bp product, a smaller fragment was reproducibly and significantly co-amplified. It was cloned and sequenced, showing that it results from the splicing of intron 11 at an alternative donor site (AG-gtgat) in exon 11 located 21 bases upstream of the usual splice site (AG-gtgca). This deletion does not change the reading frame of the mRNA and is responsible, at the protein level, for the absence of a 7 amino acids long sequence explaining the name given to this variant (NRP1-Δ7). The actual existence of this variant of NRP1 was further confirmed by different independent observations. Its presence was first evaluated in various cell lines and tissues of human or mouse origin by regular RT-PCR followed by gel electrophoresis. The relative proportion of NRP1-Δ7 was also determined by RT-qPCR using variant-specific primers. The ratio Δ7/FS varies between 0.13 and 0.43 (Fig 2). As an additional approach, the existence and presence of NRP1-Δ7 was also evaluated in “Deep Sequencing” data previously obtained using MCF7 cells mRNA [20]. From such data, the abundance of alternative splicing variants can be calculated by comparing their FPKM (Fragments Per Kilobase Of Exon Per Million Fragments Mapped). Values of 29.13 and 12.18 were obtained for NRP1-FS and NRP1-Δ7, which correspond to a ratio of 29%. Finally, the existence of NRP1-Δ7 is further assessed by its identification by the German Genome Project (clone DKFZp686E15115; http://www.ncbi.nlm.nih.gov/nuccore/BX648250.1) by entries in Genbank under the accession numbers NM_001244973 (NRP1 variant 5) and NP_001231902 (NRP1 isoform E). Based on the exon content of the previously described variants of NRP1, NRP1-s12 and NRP1-ΔE16 should be also affected by the 21 bases deletion. It was confirmed for NRP1-s12 (NRP1-s12-Δ7), while it was not further investigated for NRP1-ΔE16 which is barely detectable in our cell models (S1 Fig).

### NRP1-Δ7 Displays Reduced O-Linked Glycosylation

**Fig 3:**
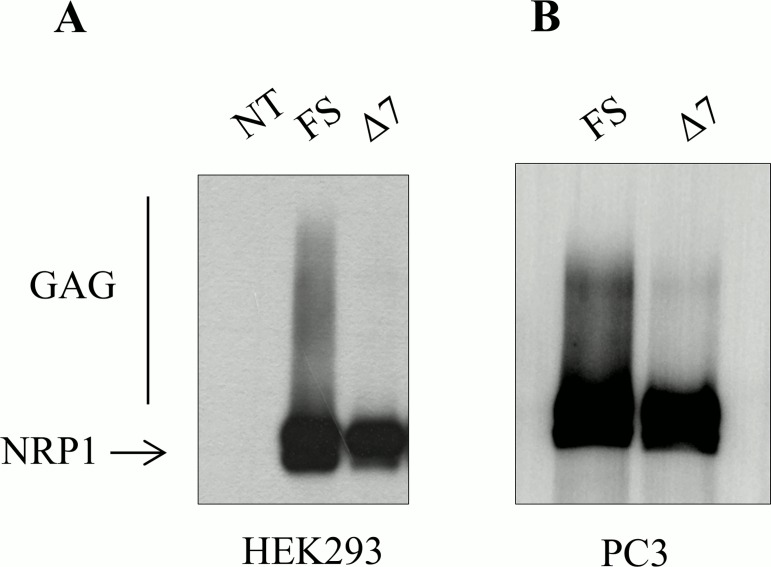
NRP1-Δ7 protein is less glycosylated than NRP1-FS protein. (A) HEK293 cells were mock-transfected (NT) or transfected with an expression vector for NRP1-FS (FS) or NRP1-Δ7 (Δ7). (B) PC3 cells were transfected with an expression vector for FLAG-NRP1-FS (FS) or for FLAG-NRP1-Δ7 (Δ7). Cells were lysed 48h after transfection and lysates were subjected to immunoprecipitation with anti-NRP1 (A) or anti-FLAG (B) antibodies. The immune-precipitates were subjected to western blotting revealed with anti-NRP1 antibodies. The smear of higher molecular weight products (GAG) corresponds to glycosaminoglycan-modified NRP1.

Recombinant proteins were produced to identify potential functional consequences of the 7 aa deletion. HEK293 cells were transiently transfected with an expression vector for NRP1-FS or NRP1-Δ7, while PC3 cells were transfected with a vector expressing FLAG-tagged NRP1-FS or NRP1-Δ7 to allow discriminating exogenous and endogenous NRP1. They were immune-precipitated, respectively, with anti-NRP1 and anti-FLAG antibodies and subjected to western blot analysis revealed by anti-NRP1. As illustrated in Fig 3, a substantial fraction of NRP1-FS migrated as high molecular weight products forming a smear corresponding to proteoglycan-modified NRP1 [10, 12]. Remarkably, these high molecular weight products were far less abundant in NRP1-Δ7 overexpressing cells, both in HEK and PC3 cells. These results suggest that the 7 aa deletion close to Ser_612_ affects its glycosylation because it changes the consensus sequence required for optimal O-glycosylation. These results also show that our cell models are adequate for investigating the biological functions of NRP1-Δ7. To improve the significance of the results of our biological models and avoid potential off-target effects linked to the use of different clones, we then generated inducible clones overexpressing NRP1-FS and NRP1-Δ7 upon treatment by doxycycline.

### Generation of Inducible PC3 and MDA-MB-231 Clones Overexpressing NRP1-FS or NRP1-Δ7

**Fig 4:**
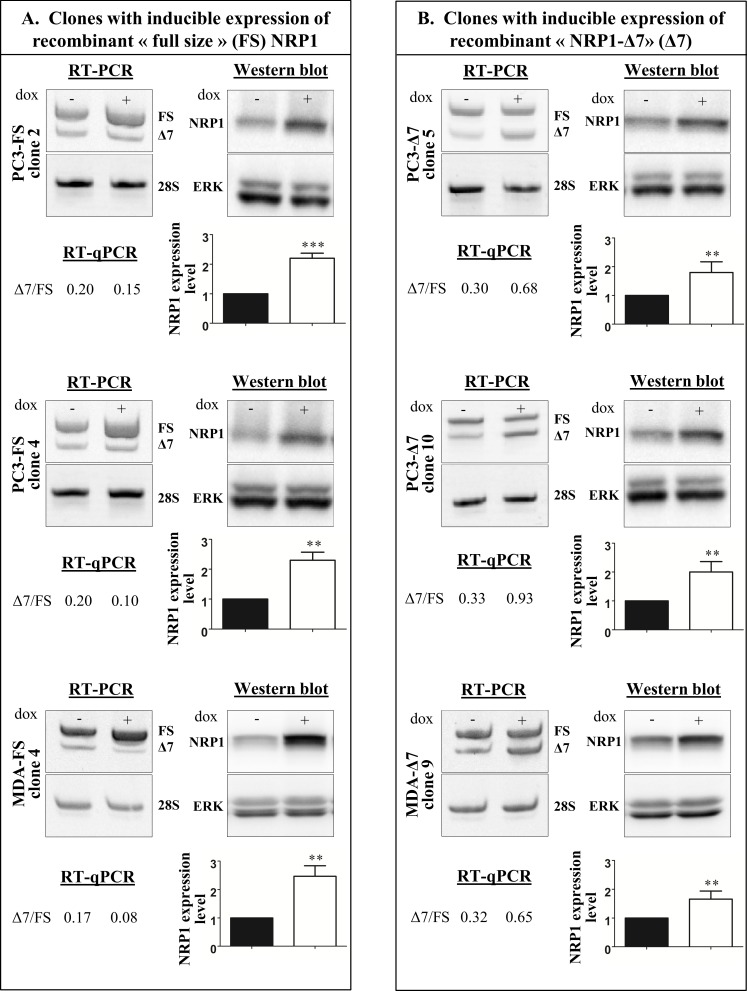
Characterization of the doxycycline-inducible clones. PC3 or MDA-MB-231 (MDA) clones with inducible expression of recombinant full size NRP1 (FS) (A) or recombinant NRP1-Δ7 (Δ7) (B) were cultured for 24h in absence (-) or presence (+) of doxycycline (dox) at 200 ng/ml. For each clone, cells were processed for total RNA extraction to perform RT-PCR and RT-qPCR analyses or lysed for western blotting visualization using an anti-NRP1 antibody. PC3 and MDA-MB-231 cells express endogenous NRP1-FS and NRP1-Δ7, which can be identified as two RT-PCR products in absence of dox. The ΔCt of each isoform were measured by SYBR green RT-qPCR and the relative expression of NRP1-FS and NRP1-Δ7 were calculated as described in Material and Methods. Induced expression of recombinant NRP1 by dox increases the relative proportion of NRP1-FS (A) or NRP1-Δ7 (B) in the corresponding clones. Due to the small difference in size (7 amino acids), NRP1-FS and NRP1-Δ7 cannot be discriminated by western blotting. Increases in total NRP1 protein expression is however observed in presence of dox. 28S rRNA and ERK1/2 were used as control for, respectively, RT-PCR and western blotting analysis.

PC3 and MDA-MB-231 cancer cells are known to constitutively express a significant level of NRP1 and to respond to NRP1-mediated signaling [22–24]. In order to benefit from a robust experimental model, we decided to create inducible clones expressing recombinant NRP1 (either FS or Δ7) only upon stimulation by doxycycline added in the culture medium. The advantage of this model is that each clone can be used as its own control by comparing its phenotype in absence and presence of inducer, an experimental setting much more reliable and biologically relevant than the comparison of different cell lines. We generated doxycycline-inducible PC3 and MDA-MB-231 clones overexpressing NRP1-FS (PC3/TR/NRP1-FS and MDA/TR/NRP1-FS) or NRP1-Δ7 (PC3/TR/NRP1-Δ7 and MDA/TR/NRP1-Δ7). Two clones of each type for PC3 and one clone of each type for MDA-MB-231 were isolated and amplified for further investigations. In control conditions without doxycycline the ratio Δ7/FS represents roughly 0.17 to 0.33 (Fig 4 and S3 Fig). Upon stimulation of expression of recombinant NRP1 by doxycycline, this ratio drops to 0.10 in NRP1-FS clones and increases up to 0.60–0.90 in NRP1-Δ7 clones, as measured by RT-qPCR. Western blotting analysis, which cannot discriminate the two isoforms, indicates a maximum of 2-fold increase in total NRP1 protein expression.

### NRP1-FS and NRP1-Δ7 Differentially Regulate Cellular Functions *In Vitro*

**Fig 5:**
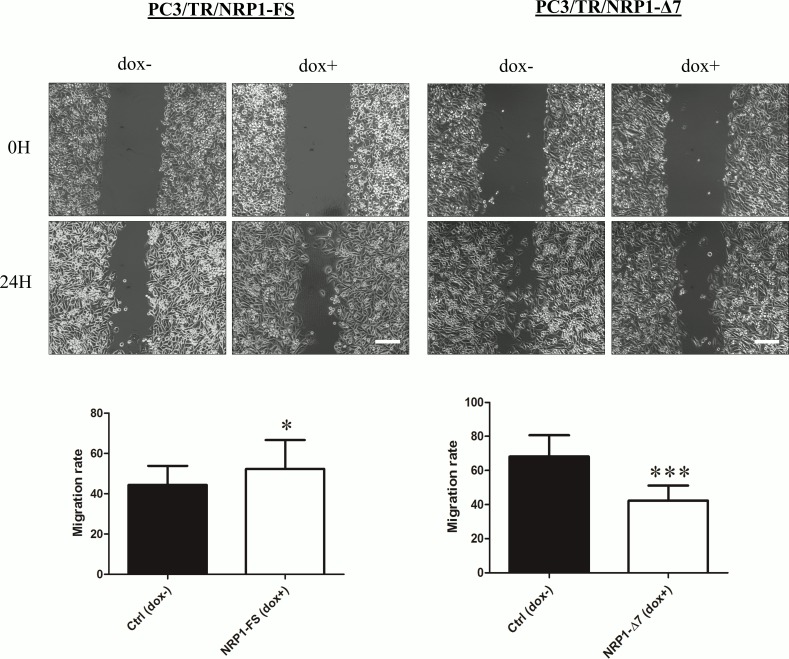
NRP1-Δ7 inhibits migration of PC3 cells. A wound scratch assay was performed according to the procedure described in Materials and Methods using the doxycycline-inducible PC3 cells overexpressing NRP1-FS (PC3/TR/NRP1-FS) or NRP1-Δ7 (PC3/TR/NRP1-Δ7) with (dox+) or without (dox-) doxycycline. Pictures were taken at the start of the experiment (0h) and 24 hours later (24h). Pictures of representative experiments are shown and the means ± SD are illustrated in the graphs (n = 12, for each cell line; *: p<0.05 and ***: p<0.001, Mann-Whitney U test)—Scale bar = 200 μm.

**Fig 6:**
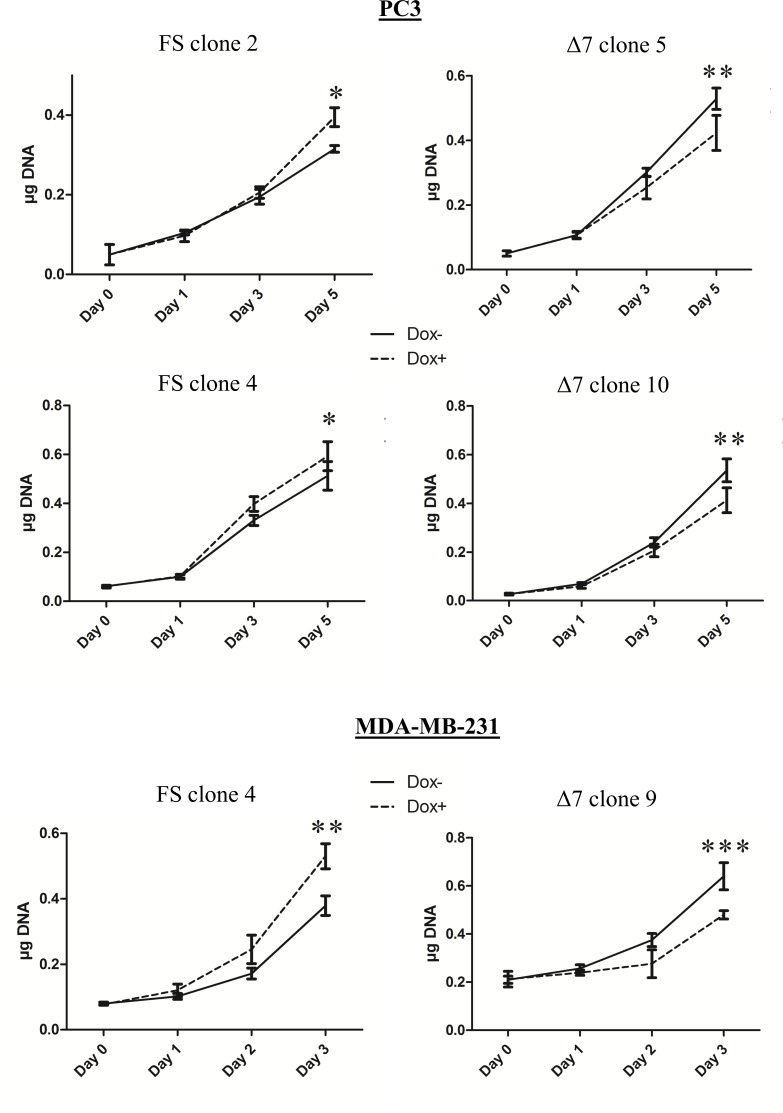
NRP1-Δ7 reduces cancer cell proliferation *in vitro*. Doxycycline-inducible PC3 and MDA-MB-231 cells able to express recombinant NRP1-FS (FS) or NRP1-Δ7 (Δ7) were seeded in 24-well plates in presence (dotted lines) or absence (continuous lines) of 200 ng/ml doxycycline and collected at the indicated times. The DNA content of each well was measured as described in Materials and Methods (n = 4, *: p<0.05, **: p<0.01 and ***: p< 0.001 as determined by Anova).

**Fig 7:**
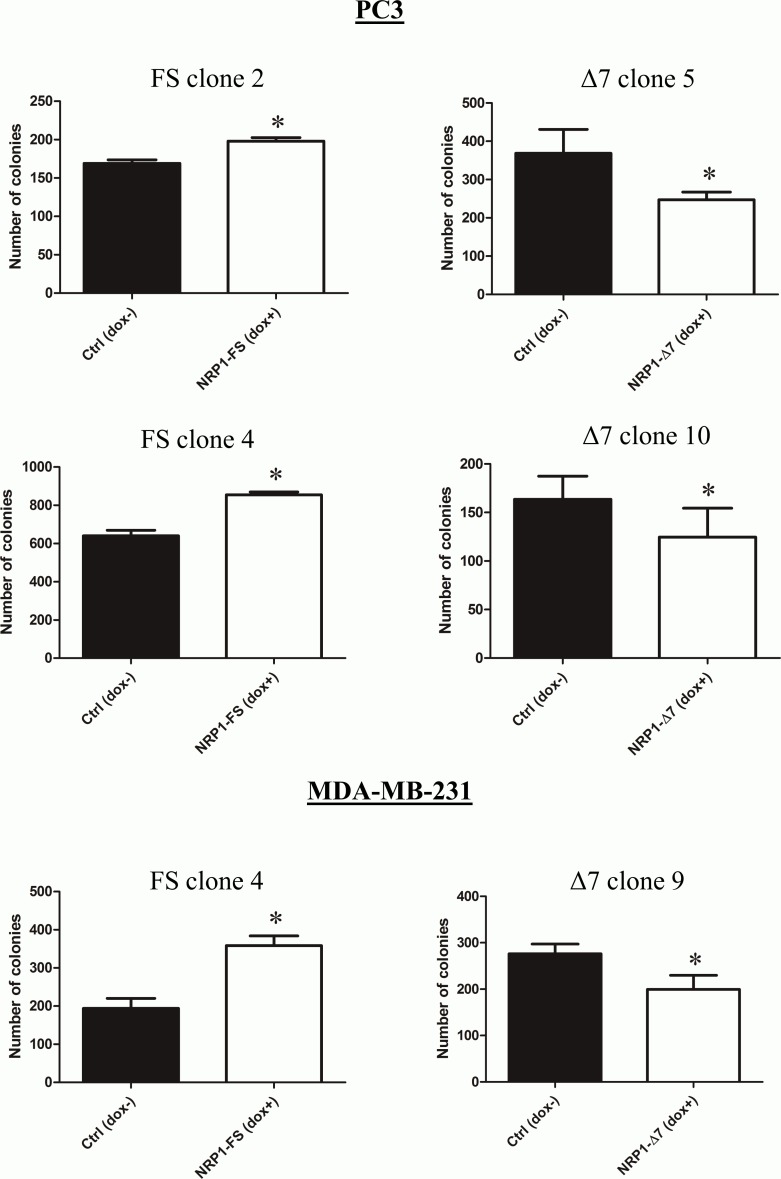
Overexpression of NRP1-Δ7 inhibits anchorage-independent growth of PC3 and MDA-MB-231 cells. Doxycycline-inducible PC3 and MDA-MB-231 clones able to express recombinant NRP1-FS (FS) or NRP1-Δ7 (Δ7) were plated in soft agar as described in Materials and Methods. Cells were supplemented (dox+) or not (dox-) with weekly renewed doxycycline (1 μg/ml). After 3 weeks of culture, colonies were counted in the whole dishes. Results are represented as the number of colonies expressed as mean ± SD. Representative results of two independent experiments performed in quadruplicates are shown (*: p<0.05, as determined by Mann-Whitney U test).

It is known that NRP1 can affect various cellular functions, such as migration or proliferation, which are relevant to cancer progression [25]. The migration of PC3 cells conditionally overexpressing NRP1-FS or NRP1-Δ7 was assessed in a scratch wound healing assay (Fig 5). As expected, overexpression of NRP1-FS significantly enhanced the migration rate of PC3 cells whereas it was significantly inhibited in cells overexpressing NRP1-Δ7. Similar results were obtained with a second PC3 clone of each type (NRP1-FS or NRP1-Δ7) and confirmed in MDA-MB-231 clones, showing that this regulation is not limited to a single cell type (S5 Fig). The proliferation rate of PC3 and MDA-MB-231 cells was also increased upon overexpression of NRP1-FS, while being reduced in cells overexpressing NRP1-Δ7 (Fig 6). These effects were not related to alterations of apoptotic processes which were not influenced by the overexpression of either variant as shown by flow cytometry analyses (S6 Fig). The consequences of the overexpression of NRP1-FS and NRP1-Δ7 on the *in vitro* tumorigenic properties of PC3 and MDA-MB-231 cells were further evaluated in an anchorage-independent growth model. Cells were seeded in soft agar in absence and presence of doxycycline and the total number of colonies formed after 3 weeks was counted. The overexpression of NRP1-FS in both cell types significantly increased the number of colonies while overexpression of NRP1-Δ7 had the opposite effect (Fig 7). It has to be noticed that doxycycline by itself did not affect the phenotype of PC3/TR cells transfected with an empty pcDNA4/TO or of MDA-MB-231/TR in the three experimental models, confirming that the observed effects are specifically related to the transgene overexpression (S4 Fig). Altogether, these data demonstrate an opposite effect of NRP1-FS and NRP1-Δ7 on the *in vitro* tumorigenic properties of PC3 and MDA-MB-231 cells.

### NRP1-FS and NRP1-Δ7 Are Differentially Relocalized upon VEGF-A_165_ Stimulation

**Fig 8:**
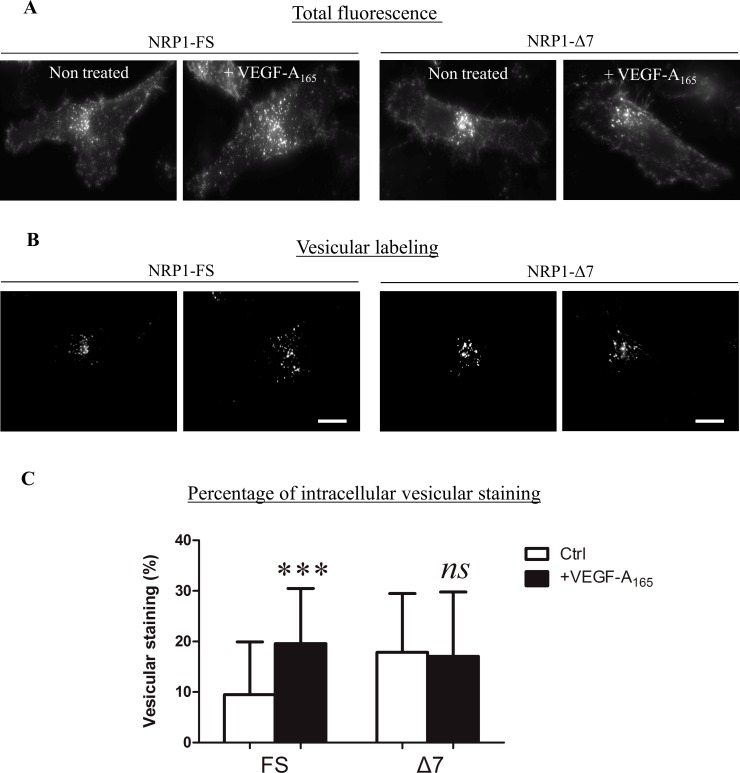
NRP1-FS and NRP1-Δ7 are differentially relocalized after VEGF-A_165_ stimulation. PC3 clones induced (dox at 200 ng/ml) to express FLAG-tagged NRP1-FS or NRP1-Δ7 were serum-starved for 24h before exposure or not to VEGF-A_165_ for 10 minutes. Immunofluorescence analyses were performed using a FLAG antibody and imaging parameters were set to visualize (A) the total amount of tagged-NRP1 or (B) only NRP1 associated with intracellular vesicles in the same cells as in (A). Quantification of the intracellular vesicular NRP1 was performed as described in Materials and Methods. Results are expressed as the mean ± SD (n = 69, FS, 0 VEGF; n = 110, FS +VEGF; n = 77 Δ7, 0 VEGF and n = 72, Δ7, +VEGF; ***: p<0.001, Mann-Whitney U test)—Scale bar = 10 μm.

NRP1 is a well-known co-receptor for VEGF. PC3 inducible clones expressing the FLAG-tagged NRP1-FS or NRP1-Δ7 were cultured in presence of doxycycline and exposed to VEGF-A_165_ for 10 min. Immunofluorescence labeling was performed with an anti-FLAG antibody and the fluorescence associated with the membrane and intracellular vesicles was quantified on Z stacked sections as detailed in Materials and Methods. As illustrated in Fig 8, VEGF-A_165_ treatment induced a significant relocalization of NRP1-FS in intracellular vesicles with a concomitant reduction of membrane-associated fluorescence. No significant difference in the compartmentalization of the NRP1-Δ7 was observed after VEGF-A_165_ treatment.

### NRP1-Δ7 Antagonizes Tumor Growth and Vascularization *In Vivo*

**Fig 9:**
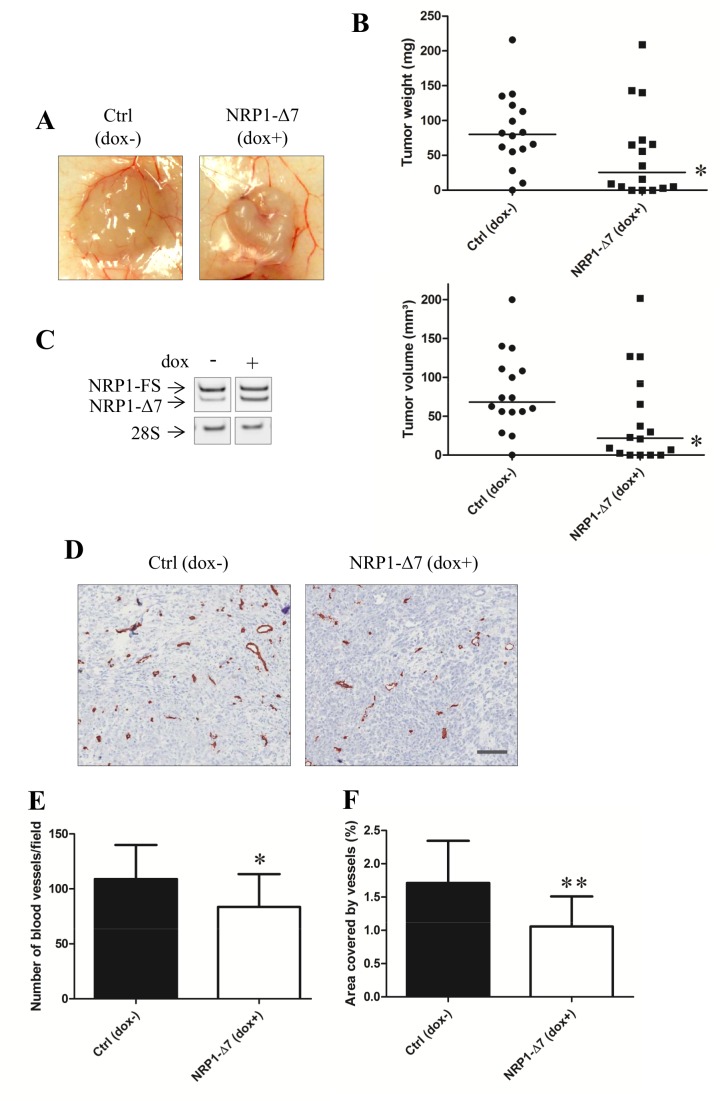
NRP1-Δ7 inhibits tumor growth and vascularization *in vivo*. (A) 2 x 10^6^ PC3/TR/ NRP1-Δ7 cells were injected in each flank of nude mice (n = 8 mice). The animals were separated in two groups. The expression of the transgene was induced in one group by adding doxycycline (2 mg/ml) in drinking water. After 7 weeks, mice were sacrificed. The tumors were macroscopically observed (A), measured and weighed (B) (n = 15 tumors in control condition and n = 14 tumors in doxycycline-induced mice) and processed for total RNA extraction and measurement of both variants by RT-PCR (C). The relative proportion of NRP1-Δ7 increases from 20 to 50% in presence of dox. (D) Immunological analysis of tumor vascularization by CD31 immunostaining. (E) The number of blood vessels per unit surface was determined and (F) the surface covered by blood vessels was calculated and expressed in %. Results are expressed as the mean ± SD of n = 13 tumors in control condition and n = 10 tumors in doxycycline-induced mice (*: p<0.05, Mann-Whitney U test)—Scale bar = 100 μm.

For evaluating the role of NRP1-Δ7 in *in vivo* tumorigenesis, PC3/TR/NRP1-Δ7 cells were injected in the flank of nude mice. Control mice had access to regular water while the water of NRP1-Δ7 mice was supplemented with doxycycline. After 7 weeks, mice were sacrificed and the tumors weighed (Fig 9A–9C). Expression of NRP1 in tumors was determined by RT-PCR. In control tumors, NRP1-Δ7 represents roughly 20% of the total amount of NRP1, while this proportion rises to 50% upon doxycycline treatment, as also observed in *in vitro* experiments. A significant inhibition of growth was observed in tumors formed by NRP1-Δ7 overexpressing cells as compared to the tumors formed by the same pool of cells in absence of doxycycline. Tumors vascularization was evaluated by labeling with anti-CD31 antibodies (Fig 9D–9F). A significantly decreased number of intratumoral blood vessels per unit surface as well as a reduction of the surface covered by blood vessels was observed in tumors overexpressing NRP1-Δ7.

## Discussion

The neuropilin-1 pre-mRNA is alternatively spliced in several mature mRNA encoding proteins with various, sometimes opposite, properties. The full size NRP1 is anchored in the cell membrane, functions as a co-receptor for multiple ligands such as VEGF-A_165_, TGF-β and semaphorinA, and forms complexes with their signaling receptors. NRP1 is also involved in endocytosis and initiates intracellular trafficking of a number of membrane receptors. The soluble isoforms of NRP1 (NRP1-s11 and -s12) act in several settings as antagonists of neuropilin-1 signaling, mainly by competitive binding to the ligands [16]. Here we report the characterization of a novel variant (NRP1-Δ7) which differs from the full size form by the absence of a 7 amino acids sequence (GDDFQLT) located 2 amino acids downstream of Ser_612_, the main O-glycosylation site on NRP1. We have shown that NRP1-Δ7 is significantly and differently expressed in various cell types of human and mouse origin, varying between 10 and 30% of total NRP1, which suggests a specific physiological function. The ability of cells to add glycosaminoglycan chains strongly depends upon the amino acids sequence around the glycosylation site [26]. For instance, Zhang and colleagues showed that a serine-glycine peptide flanked by acidic residues favored the addition of heparan sulfate [18]. In NRP1-FS, the sequence around Ser_612_ (S_612_GTGDDF) contains two “D” acidic residues, while the “GDDFQLT” deletion in NRP1-Δ7 results in a more hydrophobic consensus sequence (S_612_GTGGTT) which is likely responsible for the defective O-glycosylation of NRP1-Δ7 reported here. Alterations of NRP1 glycosylation, induced enzymatically or by mutations of Ser_612,_ profoundly alter cancer cells phenotype [10, 13]. The role of the naturally occurring NRP1-Δ7 was therefore evaluated in functional assays relevant to cancer biology. For this purpose, prostate (PC3) and breast (MDA-MB-231) adenocarcinoma cells were used as models since they express a significant level of endogenous neuropilin-1 and are known to respond to activation of neuropilin-1-dependent pathways [22–24]. They were modified to conditionally express either recombinant NRP1-FS or NRP1-Δ7 upon doxycycline treatment. As a consequence, the relative proportion of NRP1-Δ7, which is around 20% in control cells, is decreased to about 10% in doxycycline-induced NRP1-FS while it reaches almost 50% in doxycycline-treated NRP1-Δ7 clones. It has to be pointed out that, in our inducible model, the total amount of expressed NRP1 is never increased by more than about two fold, which is important to prevent non-relevant effects that could be induced by an excessive overexpression of recombinant proteins.

NRP1-FS is known to regulate cell functions involved in cancer progression such as proliferation, migration or anchorage-independent growth [9, 13, 27]. However, all the previous studies related to endogenous NRP1 did not consider separately NRP1-FS and NRP1-Δ7. The observed effects could thus reflect the net results of a balance between two NRP1 isoforms with opposite functions. By specifically evaluating the functions of NRP1-Δ7, we demonstrated here that its overexpression to the same level as endogenous NRP1-FS was sufficient to significantly decrease the proliferation rate, the migration and the anchorage-independent growth of prostatic and breast cancer cells, pointing to an anti-tumorigenic potential of NRP1-Δ7. It is well known that NRP1 forms clusters with various growth factor receptors like VEGFR and c-Met, or plexins, and serves as a guide during their intracellular recycling [28]. It was also shown that VEGF-A and TGF-β can markedly affect the phenotype and aggressiveness of PC3 cells [24]. By analyzing the subcellular dynamics of NRP1-FS and NRP1-Δ7, we showed that NRP1-Δ7 is less internalized than NRP1-FS upon VEGF-A binding. These results suggest that the association of NRP1-Δ7 with transmembrane growth factor receptors could affect their intracellular recycling which might inhibit their downstream signaling pathways. Alternatively, NRP1-Δ7 could exert its anti-tumorigenic activity by competing with NRP1-FS for binding to ligands as reported for the soluble forms of NRP1. NRP1-Δ7 could also mediate its anti-tumorigenic effects via cell adhesion receptors like integrins. NRP1 can indeed synergize with these extracellular matrix receptors to promote cancer cell proliferation, migration or colony formation in soft agar *in vitro* [9, 13, 27] and to stimulate *in vivo* tumor growth and vascularization [29]. The potential anti-tumorigenic properties of NRP1-Δ7 were therefore evaluated *in vivo* by using inducible PC3/TR/NRP1-Δ7 in a xenograft model. As observed *in vitro*, the addition of doxycycline in drinking water allowed continuous expression of NRP1-Δ7 in the xenografts. Using this model, we clearly demonstrated that increased expression of NRP1-Δ7 inhibits tumor growth and intratumoral angiogenesis.

This report is the first study characterizing the specific functions of NRP1-Δ7, a glycosaminoglycan-defective neuropilin-1 splice variant that displays anti-tumorigenic properties *in vitro* and *in vivo*. Although the precise mechanisms of regulation depending on NRP1-Δ7 were not identified, our observations emphasize the critical importance of considering specifically the different NRP1 variants, especially NRP1-Δ7, instead of analyzing the whole NRP1 expression.

## Supporting Information

S1 Fig Neuropilin-1-ΔE16 splice variant constitutes a minor fraction of the total amount of NRP1. Specific primers were designed to co-amplify the NRP1-FS and the NRP1-ΔE16 variant in PC3 and MDA-MB-231. NRP1-ΔE16 variant corresponds to 1% and 3% of NRP1-FS in, respectively, MDA-MB-231 and PC3 cells.
(TIF)

S2 Fig RT-qPCR quantification of NRP1-FS and NRP1-Δ7 expression. RT-qPCR measurements were performed on total RNA extracted from cells and tissues from human (A) and mouse (B) origin as detailed in Materials and Methods. The ΔCt and the ratio Δ7/FS were calculated as described in Materials and Methods. (C) At the end of the run, the amplification products of NRP1-FS and NRP1-Δ7 were separated by gel electrophoresis and displayed the expected size.
(TIF)

S3 Fig RT-qPCR quantification of NRP1-FS and NRP1-Δ7 expression in the doxycycline-inducible clones. PC3 or MDA-MB-231 (MDA) clones with inducible expression of recombinant full size NRP1 (FS) or recombinant NRP1-Δ7 (Δ7) were cultured for 24h in absence (-) or presence (+) of doxycycline (dox) at 200 ng/ml. For each clone, cells were processed for total RNA extraction to perform RT-qPCR analyses. The ΔCt and the ratio Δ7/FS were calculated as described in Materials and Methods.
(TIF)

S4 Fig Doxycycline by itself has no effect on the studied biological processes. Migration, proliferation and growth in soft agar assays were performed in absence and presence of doxycycline on cells transfected with an empty vector to assess the absence of influence of doxycycline by itself on these processes.
(TIF)

S5 Fig Additional clones tested in scratch wound healing assay. Additional clones of PC3 and MDA-MB-231 were used for the evaluation of the NRP1 isoform-related effects on the cell migration.
(TIF)

S6 Fig NRP1-FS or NRP1–Δ7 overexpression does not alter cell apoptosis. Apoptosis was evaluated by flow cytometry analysis of doxycycline-inducible clones of PC3 overexpressing NRP1-FS and NRP1-Δ7 after annexin V-FITC and Propidium Iodide staining as detailed in Materials and Methods.
(TIF)
