# Supplementary figures and images for: A Novel Physiological Glycosaminoglycan-Deficient Splice Variant of Neuropilin-1 Is Anti-Tumorigenic In Vitro and In Vivo
Source: PLoS One. 2016 Oct 31;11(10):e0165153. doi: 10.1371/journal.pone.0165153 (PMC5087894; doi:10.1371/journal.pone.0165153)

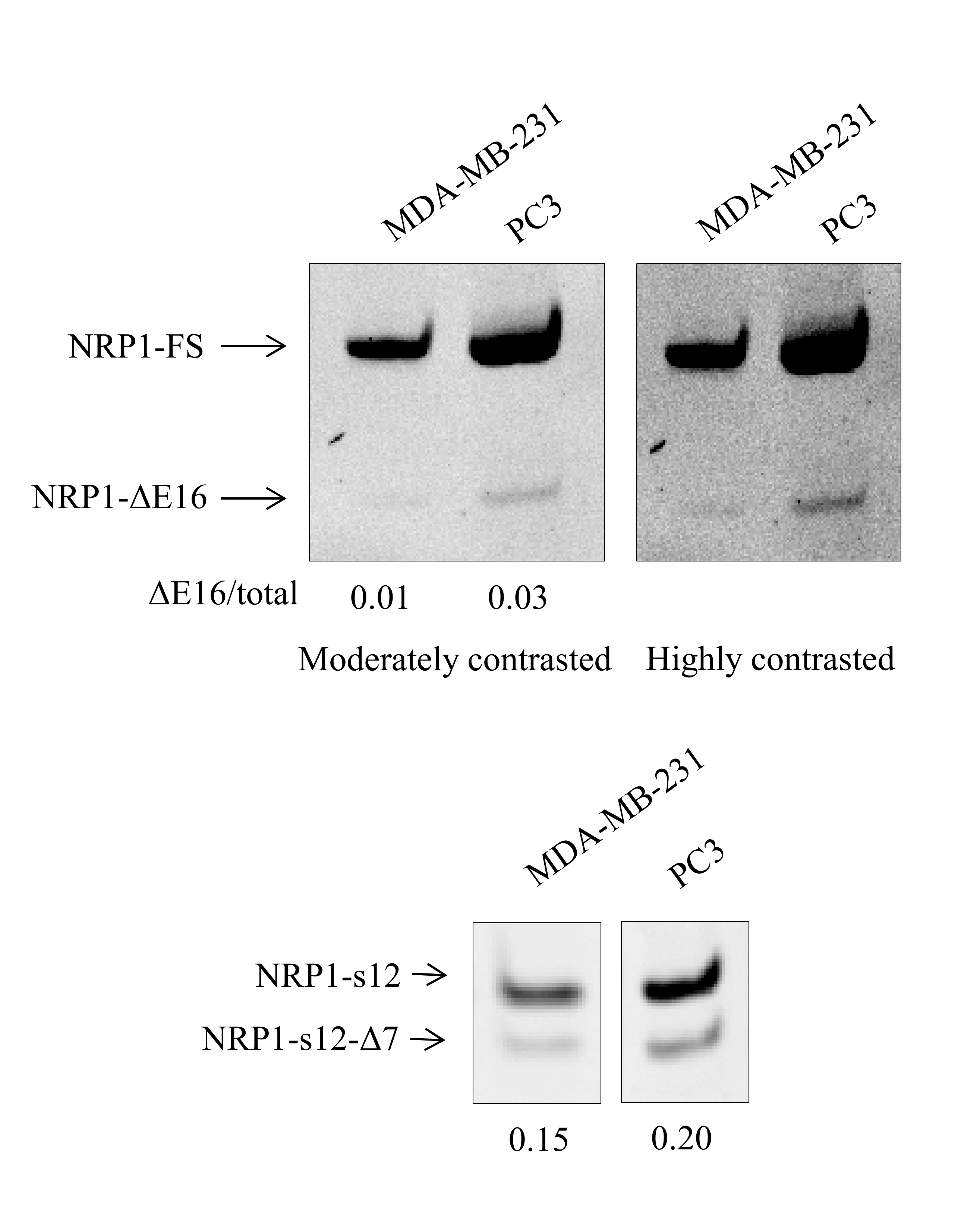

Supplement: S1 Fig — Specific primers were designed to co-amplify the NRP1-FS and the NRP1-ΔE16 variant in PC3 and MDA-MB-231. NRP1-ΔE16 variant corresponds to 1% and 3% of NRP1-FS in, respectively, MDA-MB-231 and PC3 cells. (TIF) [file pone.0165153.s001.tif]

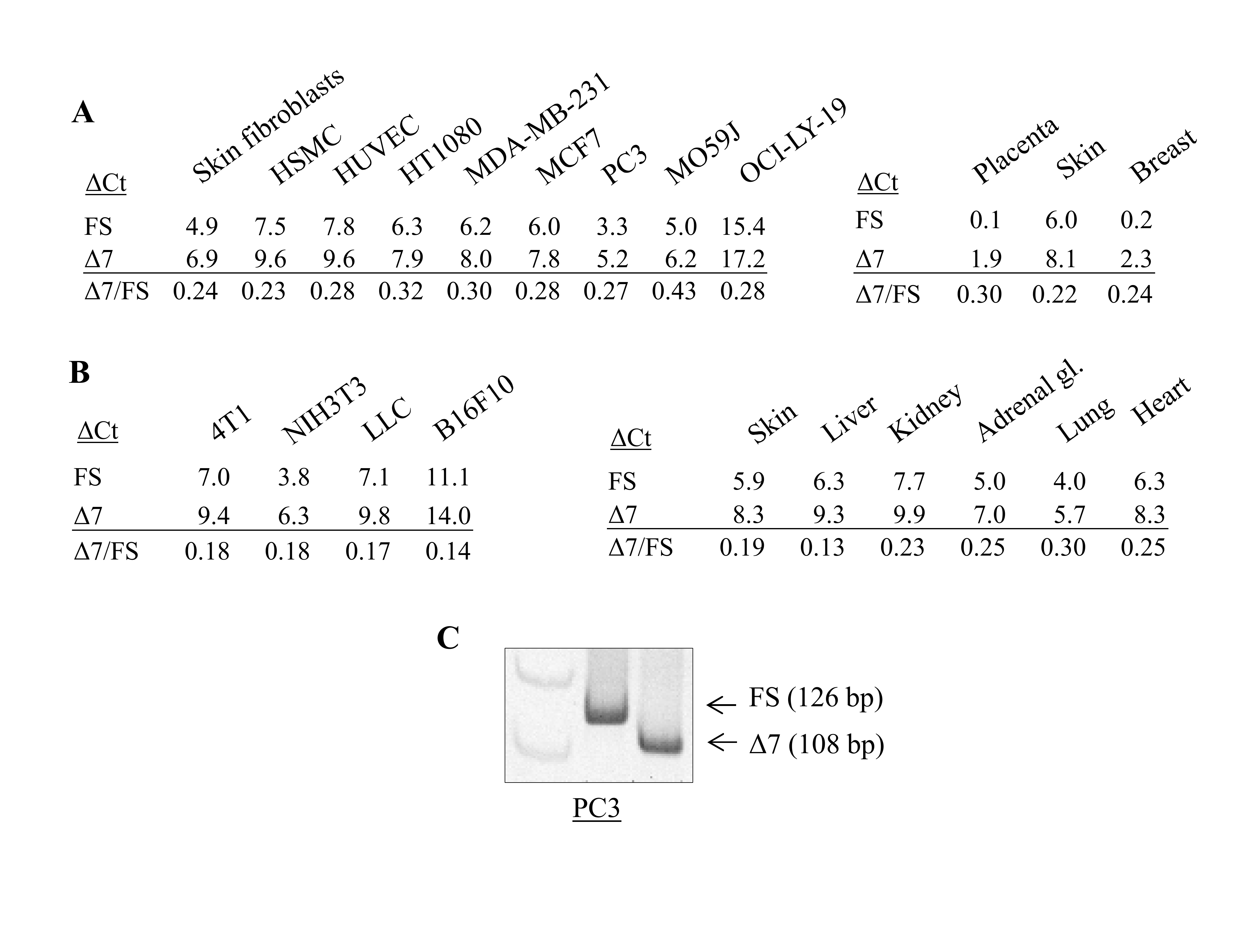

Supplement: S2 Fig — RT-qPCR measurements were performed on total RNA extracted from cells and tissues from human (A) and mouse (B) origin as detailed in Materials and Methods. The ΔCt and the ratio Δ7/FS were calculated as described in Materials and Methods. (C) At the end of the run, the amplification products of NRP1-FS and NRP1-Δ7 were separated by gel electrophoresis and displayed the expected size. (TIF) [file pone.0165153.s002.tif]

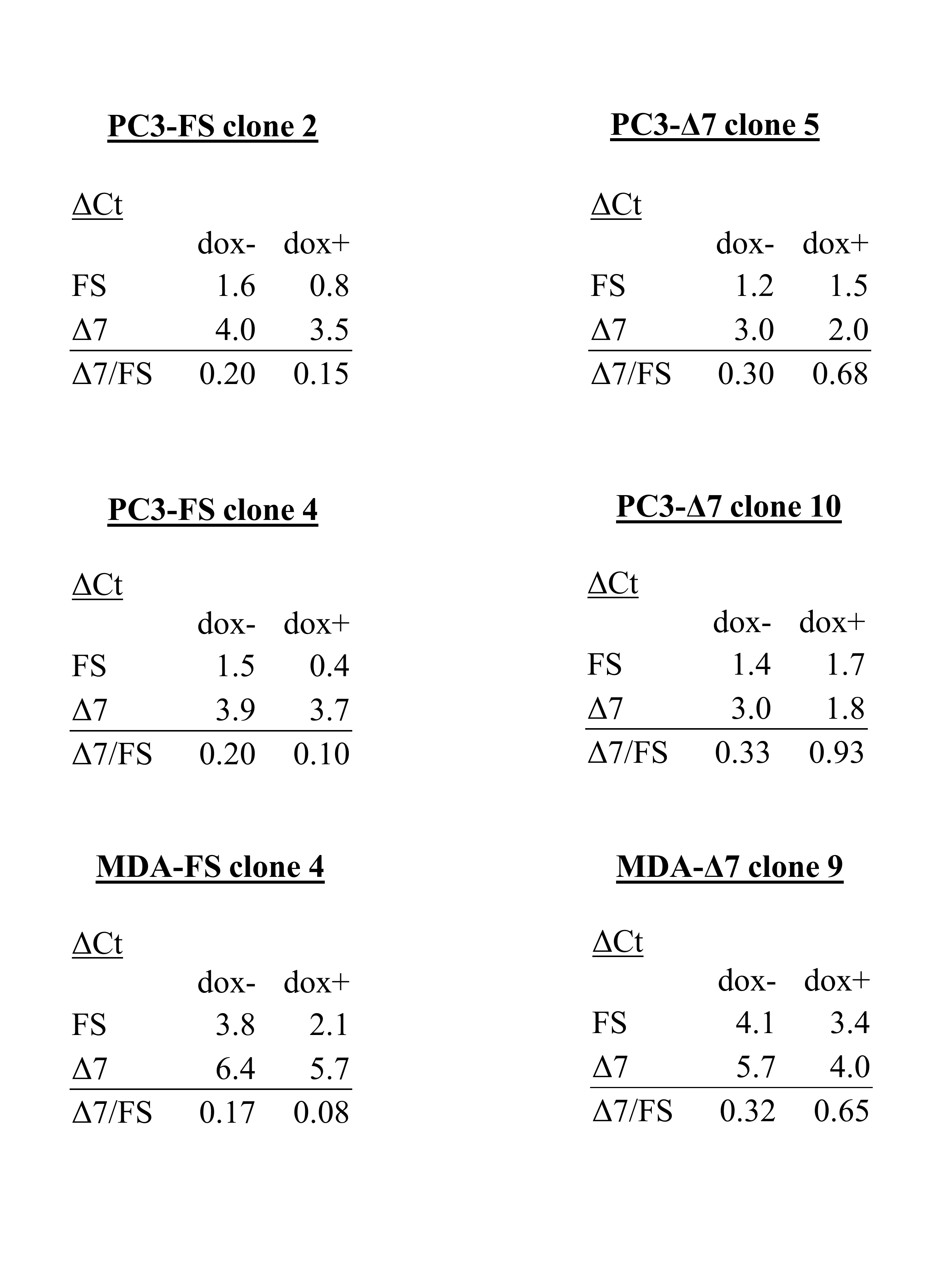

Supplement: S3 Fig — PC3 or MDA-MB-231 (MDA) clones with inducible expression of recombinant full size NRP1 (FS) or recombinant NRP1-Δ7 (Δ7) were cultured for 24h in absence (-) or presence (+) of doxycycline (dox) at 200 ng/ml. For each clone, cells were processed for total RNA extraction to perform RT-qPCR analyses. The ΔCt and the ratio Δ7/FS were calculated as described in Materials and Methods. (TIF) [file pone.0165153.s003.tif]

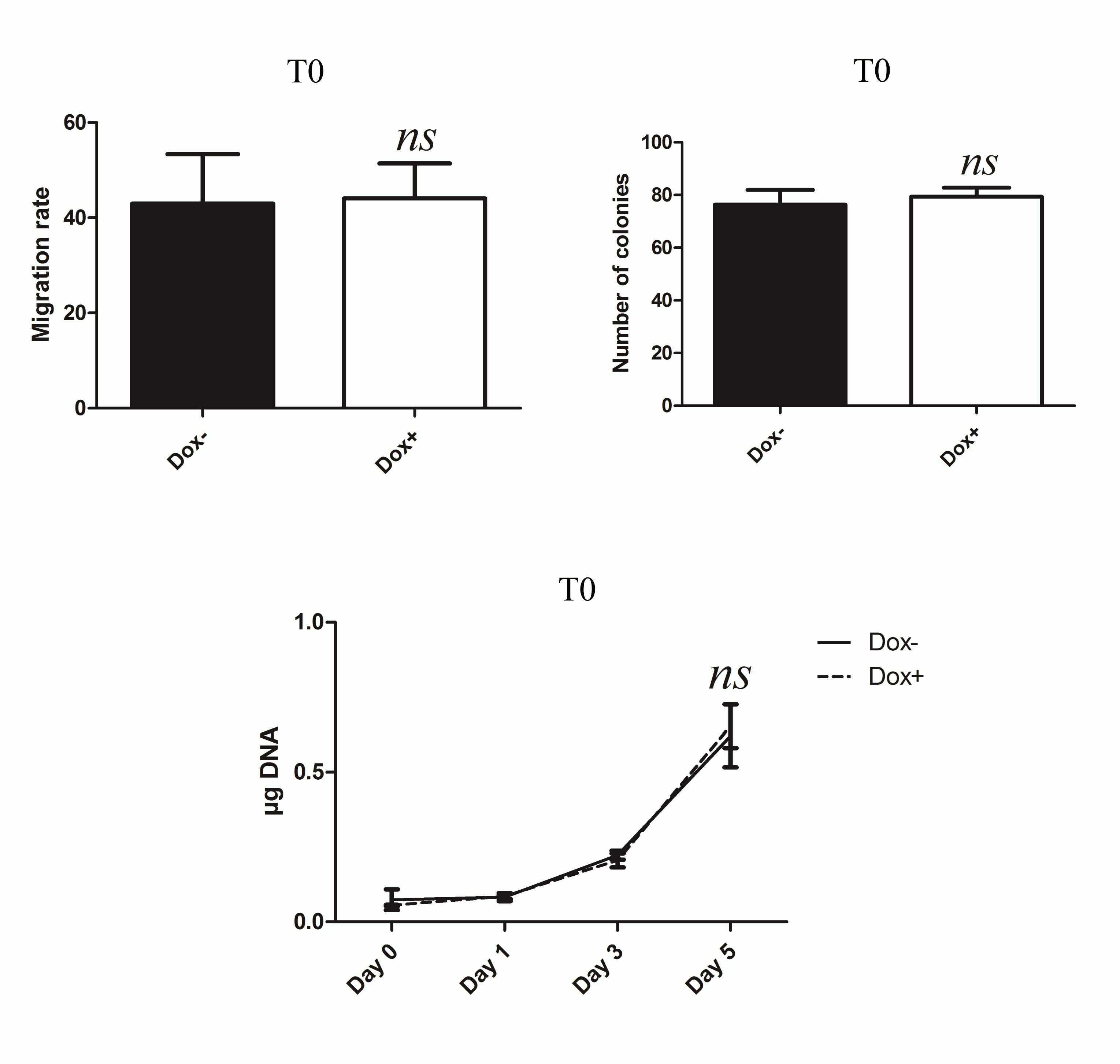

Supplement: S4 Fig — Migration, proliferation and growth in soft agar assays were performed in absence and presence of doxycycline on cells transfected with an empty vector to assess the absence of influence of doxycycline by itself on these processes. (TIF) [file pone.0165153.s004.tif]

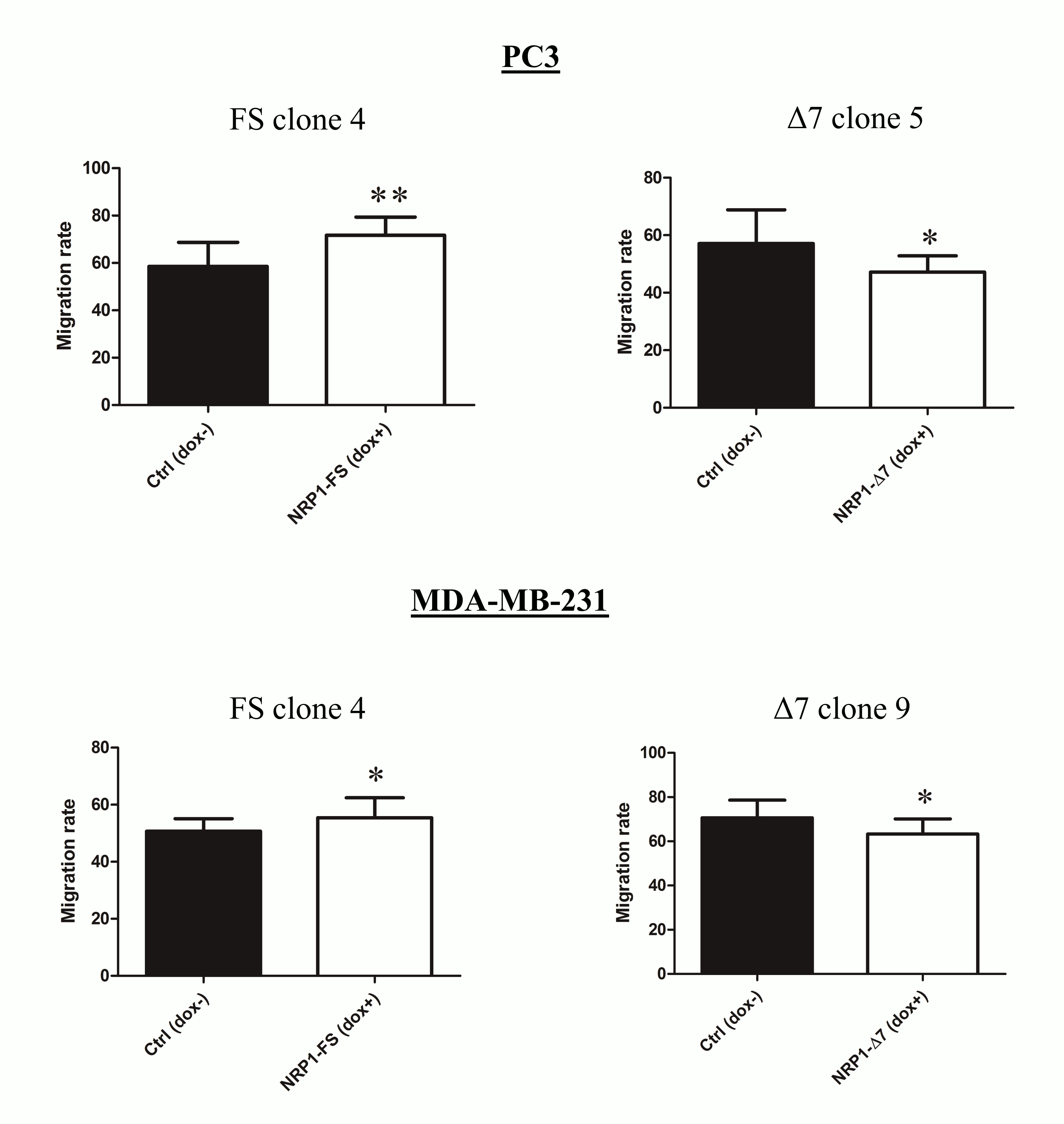

Supplement: S5 Fig — Additional clones of PC3 and MDA-MB-231 were used for the evaluation of the NRP1 isoform-related effects on the cell migration. (TIF) [file pone.0165153.s005.tif]

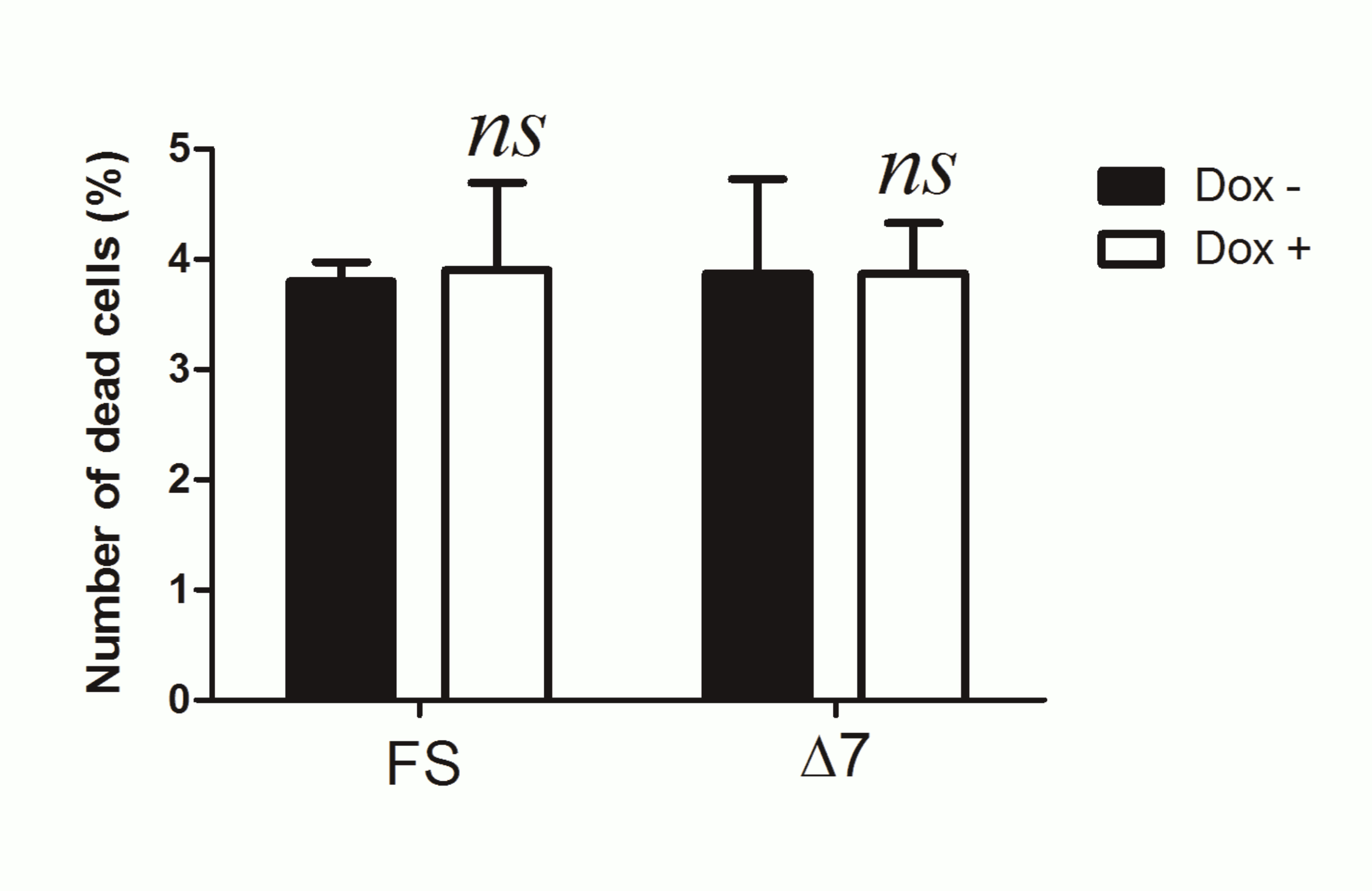

Supplement: S6 Fig — Apoptosis was evaluated by flow cytometry analysis of doxycycline-inducible clones of PC3 overexpressing NRP1-FS and NRP1-Δ7 after annexin V-FITC and Propidium Iodide staining as detailed in Materials and Methods. (TIF) [file pone.0165153.s006.tif]
